# Treatment using oxaliplatin and S-1 adjuvant chemotherapy for pathological stage III gastric cancer: a multicenter phase II study (TOSA trial) protocol

**DOI:** 10.1186/s12885-018-4109-z

**Published:** 2018-02-13

**Authors:** Tsutomu Namikawa, Hiromichi Maeda, Hiroyuki Kitagawa, Koji Oba, Akihito Tsuji, Takaki Yoshikawa, Michiya Kobayashi, Kazuhiro Hanazaki

**Affiliations:** 1 Department of Surgery, Kochi Medical School, Kohasu, Oko-cho, Nankoku, Kochi, 783-8505 Japan; 2 Cancer Treatment Center, Kochi Medical School Hospital, Kochi, Japan; 3 Department of Biostatistics, Graduate School of Medicine, The University of Tokyo, Tokyo, Japan; 4 Department of Clinical Oncology, Faculty of Medicine, Kagawa University, Kagawa, Japan; 5 Department of Gastrointestinal Surgery, Kanagawa Cancer Center Hospital, Yokohama, Japan; 6 Department of Human Health and Medical Sciences, Kochi Medical School, Kochi, Japan

**Keywords:** Gastric cancer, Adjuvant chemotherapy, Gastrectomy, Oxaliplatin, S-1, Clinical trial, Multicenter phase II study, Chemotherapy, Surgery, Survival

## Abstract

**Background:**

Recent studies demonstrated the efficacy of S-1-based adjuvant chemotherapy administered for six months after curative surgery for stage III gastric cancer; however, it is unproven whether this type of combination chemotherapy is more effective than the standard adjuvant chemotherapy of S-1 for one year.

**Methods:**

This multicenter phase II study evaluate the efficacy and safety of adjuvant chemotherapy using S-1 plus oxaliplatin followed by S-1 for up to one year for curatively resected stage III gastric cancer in patients aged over 20 years. Treatment initially comprises oral fluoropyrimidine S-1 (80 mg/m^2^) administered twice daily for the first 2 weeks of a 3-week cycle. On day 1 of a second 3-week cycle, patients will receive 100 mg/m^2^ of intravenous oxaliplatin followed by 80 mg/m^2^ of S-1 (maximum 8 cycles). Then, the patients will receive 80 mg/m^2^ of S-1 daily for 4 weeks, followed by 2 weeks of no chemotherapy. This 6-week cycle will be repeated during the first year after surgery. The primary endpoint is relapse-free survival for 3 years and secondary endpoints are safety, including the incidence of adverse events, and grading of neuropathy with each treatment cycle. The planned sample size of 75 patients is appropriate for this trial. The data will be analyzed on an intention-to-treat basis, assuming a two-sided test with a 5% level of significance.

**Discussion:**

In contrast to previous trials, the current study involves administration of S-1 until one year after surgery in addition to prior S-1 plus oxaliplatin, and is the first study to evaluate the safety and efficacy of S-1 plus oxaliplatin followed by S-1 for up to one year in patients with curatively resected stage III gastric cancer.

**Trial registration:**

This trial is registered in the University Hospital Medical Information Network’s Clinical Trials Registry (UMIN-CTR) registration number, R000029656  (https://upload.umin.ac.jp/cgi-open-bin/ctr_e/ctr_view.cgi?recptno=R000029656). Registered January 24, 2017.

## Background

Gastric cancer is the third most commonly diagnosed cancer worldwide, and one of the leading causes of cancer-related deaths [1]. Although early gastric cancer has a good prognosis with surgical resection, advanced gastric cancer shows disease recurrence at a constant rate depending on disease stage, resulting in 5-year postoperative survival rates of 70% for stage II, 47% for stage IIIA, 29% for stage IIIB, and 15% for stage IV [2]. Therefore, adjuvant chemotherapy has been widely investigated as a potentially standard component of resectable gastric cancer therapy to improve patient outcomes [3, 4].

S-1 is an orally active combination of tegafur, a prodrug that is converted by cells to fluorouracil, gimeracil, which inhibits dihydropyrimidine dehydrogenase, and oteracil, which inhibits the phosphorylation of fluorouracil in the gastrointestinal tract, thereby reducing the toxic gastrointestinal effects of fluorouracil. Postoperative adjuvant chemotherapy using S-1 for one year has become a standard treatment in patients treated by curative gastrectomy with D2 lymph node dissection for stage II or stage III gastric cancer on the basis of results from a randomized phase III study comparing surgery plus adjuvant S-1 with surgery alone (ACTS-GC trial) [3, 5]. However, the 5-year overall survival (OS) rates in stage IIIA and stage IIIB patients receiving S-1 were 67.1 and 50.2%, respectively, which are less satisfactory compared with the rate for stage II (84.2%) [5]. Therefore, patients with stage III gastric cancer are in urgent need of more effective adjuvant chemotherapy.

Recent studies demonstrated the efficacy of S-1-based adjuvant chemotherapy administered for six months after curative surgery for stage III gastric cancer, including S-1 plus cisplatin or S-1 plus oxaliplatin [6–8]; however, it is unproven whether this type of combination chemotherapy is more effective than the standard adjuvant chemotherapy of S-1 for one year. This multicenter phase II study evaluated the safety and efficacy of S-1 plus oxaliplatin for patients with curatively resected stage III gastric cancer.

## Methods

### Study objective

The trial is conducted in accordance with the World Medical Association Declaration of Helsinki and Japanese Ethical Guidelines for Medical and Health Research Involving Human Subjects. This prospective phase II trial is planned to elucidate the efficacy and safety of adjuvant chemotherapy using S-1 plus oxaliplatin for Stage III gastric cancer. The trial protocol has been approved by the Institutional Review Board (IRB) of each participating institution and the Kochi Medical School Hospital. The protocol of this study has been registered in the University Hospital Medical Information Network’s Clinical Trials Registry (UMIN-CTR) registration number, R000029656 (https://upload.umin.ac.jp/cgi-open-bin/ctr_e/ctr_view.cgi?recptno=R000029656).

### Study endpoints

The primary endpoint is relapse-free survival (RFS), and the secondary endpoints are overall survival (OS) and safety (the incidence of adverse events and grading of neuropathy in each treatment cycle).

### Eligibility criteria

Patients with resectable gastric cancer diagnosed as pathological stage III according to the Japanese Classification of Gastric Carcinoma [9] who satisfy the inclusion criteria and do not meet the exclusion criteria as described below will be recruited as subjects.

### Inclusion criteria

1. Histologically proven, Stage III resectable gastric adenocarcinoma according to the Japanese Classification of Gastric Carcinoma.
2. D2 or more extensive lymph-node dissection with R0 surgery (with the result of no residual tumor)
3. No hepatic, peritoneal, or distant metastasis; no tumor cells in peritoneal fluid on cytological analysis.
4. Age of 20 years and over.
5. Performance status of 0–1 according to the Eastern Cooperative Oncology Group (ECOG) scale.
6. Patients who received surgery during the previous 6 weeks, being possible per-oral administration.
7. No previous treatment for cancer except for the initial gastric resection to remove the primary lesion.
8. Adequate organ function during the previous 14 days.
9. All patients agree to participate in the clinical study through informed consent.

### Exclusion criteria

1. Synchronous or metachronous malignant disease, or multicentric cancer.
2. Serious postoperative morbidity such as serious infection, anastomotic leakage, or gastrointestinal bleeding.
3. Serious concurrent disease such as intestinal palsy, small bowel obstruction, interstitial pneumonia, pulmonary fibrosis, uncontrollable diabetes mellitus, heart failure, renal failure, liver cirrhosis, or hepatic failure.
4. Obvious active infectious disease.
5. Positive for hepatitis B surface antigen or hepatitis C virus antibody.
6. Continuous treatment with steroids.
7. Serious diarrhea.
8. A medical history of serious allergy to any drug.
9. Ascites beyond the pelvic cavity or pleural effusion.
10. Clinical suspicion or previous history of metastasis to brain or meninges.
11. Taking flucytosine or phenytoin.
12. Clinical indication for warfarin potassium.
13. Sensory neuropathy caused by any drug.
14. A history of blood transfusion or administration of granulocyte colony-stimulating factor within 3 weeks before enrollment.
15. Women who are pregnant or hope to become pregnant during the study period.
16. Patients judged inappropriate for the study by their physicians.

### Treatment methods

S-1 will be given orally twice daily for the first 2 weeks of a 3-week cycle, at a dosage of 80 mg/day for a body surface area (BSA) less than 1.25 m^2^, 100 mg/day for BSA ranging from 1.25 m^2^ to less than 1.5 m^2^, and 120 mg/day for BSA of 1.5 m^2^ or greater. On day 1 of the second 3-week cycle, patients will receive 100 mg/m^2^ of intravenous oxaliplatin followed by 80 mg/m^2^ of S-1 (for a maximum of 8 cycles). Then, the patients will receive 80 mg/m^2^ of S-1 daily for 4 weeks, followed by 2 weeks of no chemotherapy. This 6-week cycle will be repeated during the first year after surgery.

### Registration

Eligible patients will be centrally registered within 42 days of surgery. Physicians or coordinators will send a Case Registration Form to the registration center, located at the Cancer Treatment Center, Kochi Medical School Hospital, with all the required items filled out. Accrual of patients began in March 2017.

### Statistical analysis

The primary endpoint of RFS is defined as the length of time from the surgery to the date that the patient survives without any signs or symptoms of that recurrence, whichever is sooner. The primary objective of this trial is to evaluate the RFS of using S-1 plus oxaliplatin as the adjuvant chemotherapy for resected stage III gastric cancer. The three years RFS will be calculated point estimates and 2-sided 90% confidence intervals. Cumulative RFS curves will be constructed as time-to event plots by the Kaplan-Meier method. With regard to secondary endpoints, OS, which is defined as the time from the date of surgery to date of death from any cause, will be evaluated according to the method of analysis of the primary endpoint. For the analysis of safety, Fisher’s exact test will be used if necessary, and the exact 95% confidence intervals for the binomial distribution will be estimated.

### Sample-size calculation

According to the ACTS-GC trial, 3-year RFS in patients with stage III gastric cancer who underwent adjuvant chemotherapy using S-1 was 62.3% (68.0% in stage IIIA and 49.8% in stage IIIB) [3]. Survival analysis of adjuvant chemotherapy with S-1 plus cisplatin for stage III gastric cancer demonstrated 74.1% RFS [8]. Under the hypothesis of a threshold 3-year RFS of 62% and an expected 3-year RFS of 75%, at 3 years follow-up after registration, 62 patients in total are needed to ensure an 1-sided alpha value of 0.05, and statistical power of 80% [10]. Considering the likelihood of some ineligible cases in the whole setting outlined above, the total sample size is set to 75.

### Follow-up

During treatment under this protocol, patients will undergo a physical check-up and a blood examination before every drug administration. During and after finishing the protocol treatment, patients are physically examined for recurrence, and tumor markers (CEA, CA19–9, CA125, and CA72–4) are measured every 3 months for 3 years. Abdominal computed tomography scans and chest radiographs are obtained every 3 months for 3 years. Adverse events will be graded according to the National Cancer Institute’s Common Terminology Criteria for Adverse Events (version 4.0). Adverse events will be documented during chemotherapy and for 28 days after the last dose of study medication. Registered patients will be followed up to confirm the presence or absence of recurrence and survival status for 5 years after accrual of the last patient.

## Discussion

Chemotherapy using S-1 plus oxaliplatin has showed promising efficacy against advanced gastric cancer in several large trials [11–13]. This trial is a multicenter prospective phase II study to evaluate the efficacy and safety of adjuvant chemotherapy using S-1 plus oxaliplatin followed by S-1 monotherapy for up to one year for curatively resected stage III gastric cancer. A recent study suggested that an 8-week cycle of adjuvant chemotherapy using S-1 and oxaliplatin was manageable and safe with optimal dose reduction and delay in 62 patients with stage III gastric cancer [6]. In contrast to this previous trial, the current study involves administration of S-1 until one year after surgery in addition to prior S-1 plus oxaliplatin, and is the first study to evaluate the safety and efficacy of S-1 plus oxaliplatin followed by S-1 for up to one year in patients with curatively resected stage III gastric cancer.

The ACTS-GC trial demonstrated that the addition of S-1 improved the OS rate, with a low incidence of adverse events and good compliance [3], while the CLASSIC trial indicated that adjuvant capecitabine and oxaliplatin improved 3-year disease-free survival compared with surgery alone in gastric cancer patients [4]. Studies in Japan also showed the feasibility of adjuvant chemotherapy using capecitabine and oxaliplatin in patients with stage II or III gastric cancer who underwent curative gastrectomy [14]. Although adjuvant therapy with S-1 plus cisplatin is also feasible [8], S-1 plus oxaliplatin therapy has several benefits including unnecessary hydration due to low renal toxicity. However, the evidence on which postoperative adjuvant chemotherapy has been established as standard therapy for the treatment of locally advanced gastric cancer remains under debate. The results of this study will therefore play an important role in identifying more effective adjuvant chemotherapy for patients with stage III gastric cancer in the near future.

One limitation of this trial is the non-randomized study design, which has known inferential deficiencies. However, the study design is appropriate for assessing the primary endpoint of RFS rate at three years in a limited number of patients to explore putative evidence of the adjuvant chemotherapy in the postoperative patients after gastrectomy for gastric cancer.

## Abbreviations

BSA: Body surface area
ECOG: Eastern Cooperative Oncology Group
IRB: Institutional Review Board
OS: Overall survival
RFS: Relapse-free survival
UMIN-CTR: University Hospital Medical Information Network’s Clinical Trials Registry

## References

[1] Siegel RL, Miller KD, Jemal A (2017). Cancer statistics, 2017. CA Cancer J Clin.

[2] Nashimoto A, Akazawa K, Isobe Y, Miyashiro I, Katai H, Kodera Y, Tsujitani S, Seto Y, Furukawa H, Oda I, Ono H, Tanabe S, Kaminishi M (2013). Gastric cancer treated in 2002 in Japan: 2009 annual report of the JGCA nationwide registry. Gastric Cancer.

[3] Sakuramoto S, Sasako M, Yamaguchi T, Kinoshita T, Fujii M, Nashimoto A, Furukawa H, Nakajima T, Ohashi Y, Imamura H, Higashino M, Yamamura Y, Kurita A, Arai K, ACTS-GC Group (2007). Adjuvant chemotherapy for gastric cancer with S-1, an oral fluoropyrimidine. N Engl J Med.

[4] Bang YJ, Kim YW, Yang HK, Chung HC, Park YK, Lee KH, Lee KW, Kim YH, Noh SI, Cho JY, Mok YJ, Kim YH, Ji J, Yeh TS, Button P, Sirzén F, Noh SH, CLASSIC trial investigators (2012). Adjuvant capecitabine and oxaliplatin for gastric cancer after D2 gastrectomy (CLASSIC): a phase 3 open-label, randomised controlled trial. Lancet.

[5] Sasako M, Sakuramoto S, Katai H, Kinoshita T, Furukawa H, Yamaguchi T, Nashimoto A, Fujii M, Nakajima T, Ohashi Y (2011). Five-year outcomes of a randomized phase III trial comparing adjuvant chemotherapy with S-1 versus surgery alone in stage II or III gastric cancer. J Clin Oncol.

[6] Shitara K, Chin K, Yoshikawa T, Katai H, Terashima M, Ito S, Hirao M, Yoshida K, Oki E, Sasako M, Emi Y, Tsujinaka T (2017). Phase II study of adjuvant chemotherapy of S-1 plus oxaliplatin for patients with stage III gastric cancer after D2 gastrectomy. Gastric Cancer.

[7] Takahari D, Hamaguchi T, Yoshimura K, Katai H, Ito S, Fuse N, Kinoshita T, Yasui H, Terashima M, Goto M, Tanigawa N, Shirao K, Sano T, Sasako M (2011). Feasibility study of adjuvant chemotherapy with S-1 plus cisplatin for gastric cancer. Cancer Chemother Pharmacol.

[8] Takahari D, Hamaguchi T, Yoshimura K, Katai H, Ito S, Fuse N, Konishi M, Yasui H, Terashima M, Goto M, Tanigawa N, Shirao K, Sano T, Sasako M (2014). Survival analysis of adjuvant chemotherapy with S-1 plus cisplatin for stage III gastric cancer. Gastric Cancer.

[9] Japanese Gastric Cancer Association (2011). Japanese classification of gastric carcinoma: 3rd English edition. Gastric Cancer.

[10] Statistical tools in Southwest Oncology Group. Available from: https://stattools.crab.org/Calculators/oneArmSurvivalColored.html. Accessed Jan 2017.

[11] Ter Veer E, Mohammad NH, Lodder P, Ngai LL, Samaan M, van Oijen MG, van Laarhoven HW (2016). The efficacy and safety of S-1-based regimens in the first-line treatment of advanced gastric cancer: a systematic review and meta-analysis. Gastric Cancer.

[12] Yamada Y, Higuchi K, Nishikawa K, Gotoh M, Fuse N, Sugimoto N, Nishina T, Amagai K, Chin K, Niwa Y, Tsuji A, Imamura H, Tsuda M, Yasui H, Fujii H, Yamaguchi K, Yasui H, Hironaka S, Shimada K, Miwa H, Hamada C, Hyodo I (2015). Phase III study comparing oxaliplatin plus S-1 with cisplatin plus S-1 in chemotherapy-naïve patients with advanced gastric cancer. Ann Oncol.

[13] Koizumi W, Takiuchi H, Yamada Y, Boku N, Fuse N, Muro K, Komatsu Y, Tsuburaya A (2010). Phase II study of oxaliplatin plus S-1 as first-line treatment for advanced gastric cancer (G-SOX study). Ann Oncol.

[14] Fuse N, Bando H, Chin K, Ito S, Yoshikawa T, Tsuburaya A, Terashima M, Kawashima Y, Fukunaga T, Gotoh M, Emi Y, Yoshida K, Oki E, Takahashi S, Kuriki H, Sato K, Sasako M (2017). Adjuvant capecitabine plus oxaliplatin after D2 gastrectomy in Japanese patients with gastric cancer: a phase II study. Gastric Cancer.

